# Research progress on the biosynthesis and regulatory mechanisms of resveratrol in plants

**DOI:** 10.3389/fpls.2026.1778295

**Published:** 2026-05-14

**Authors:** XueFeng Bao, Xuan Dong, Lin Yang, ShengChun Li, DingWei YU, XueBo Wang, XinHong Li, HuaFang Dong

**Affiliations:** Panxi Crop lmprovement Key Laboratory of Sichuan Province, College of Agricultural Science Xichang University, Xichang, Sichuan, China

**Keywords:** biosynthetic pathway, resveratrol, signal transduction, stilbene, transcriptional regulation

## Abstract

Resveratrol, a type of important polyphenolic secondary metabolite, plays a crucial role in plant resistance to biotic and abiotic stresses. In recent years, numerous studies have emerged on its biosynthetic pathways and induction effects. However, existing reviews are mostly focused on early research and fail to cover the latest advancements. This review, based on a retrospective analysis of the effects of various inducers (including biotic factors, plant hormones and growth regulators, specific light qualities, and metal ions) on resveratrol biosynthesis, with a literature search timeframe restricted to 2010–2025 and using databases including PubMed, Web of Science, and Scopus with keywords ‘resveratrol biosynthesis’, ‘resveratrol regulatory mechanism’, ‘*STS* gene regulation’, and in combination with the latest research, focuses on analyzing: (1) the regulatory patterns of *STS* promoters by transcription factors acting alone or in interaction; (2) the regulatory roles of calcium signaling, hormone signal transduction (such as jasmonic acid, salicylic acid, and abscisic acid), and MAPK cascade reactions in resveratrol biosynthesis. This review aims to provide a new perspective for a deeper understanding of the biosynthesis and complex regulatory mechanisms of resveratrol.

## 1 Introduction

Resveratrol, as a stilbene phytoalexin, is widely distributed in plants such as grapes and pines (Dubrovina and Kiselev, 2017). Its phenolic hydroxyl groups confer strong resistance to plants, and its synthesis is significantly induced and accumulated under biotic stress (e.g., pathogens and pests) and abiotic stress (e.g., UV radiation, mechanical injury) conditions. In addition, resveratrol has also shown great potential in anti-inflammatory and anticancer fields (Liu et al., 2018).

In recent years, research on resveratrol has mainly focused on the following aspects: optimization of extraction and purification processes for resveratrol; comparison of resveratrol and its derivatives contents in different plant species (or varieties); the impact of external environmental stress (e.g., high temperature, salt stress, water stress) and exogenous inducers (e.g., UV light, exogenous hormones, pathogens) on its biosynthetic amount; evaluation of biological activity (e.g., antioxidant, disease resistance); and the transcriptional regulatory mechanisms of stilbene synthase (*STS*, also known as resveratrol synthase RS).

Many botanical reviews have elaborated on the biosynthetic pathways of resveratrol and its derivatives, as well as the regulatory effects of environmental factors and exogenous inducers on its biosynthesis (Chong et al., 2009; Dubrovina and Kiselev, 2017; Valletta et al., 2021; Marant et al., 2024). However, a review published in 2017, although attempting to integrate the transcriptional regulation and signal transduction mechanisms of its biosynthesis (Dubrovina and Kiselev, 2017), failed to cover the latest research progress due to its early publication date.

Since 2018, research progress has mainly focused on the regulatory mechanisms of the *STS* promoter, including: (1) interactions between *MYB* and other transcription factors (e.g., ERF, bHLH); (2) direct regulation by other transcription factors (e.g., bZIP, Alfin-like, BSISTER); (3) cascade regulation by kinases (e.g., CML, SnRK2, NPR, MAPK) through phosphorylation of transcription factors. This review, based on a retrospective analysis of the effects of inducers (such as biotic factors, hormones, light quality, metal ions, etc.) on resveratrol biosynthesis, integrates the above regulatory mechanisms to provide a new perspective for a deeper understanding of resveratrol biosynthesis and its complex regulatory network.

## 2 Properties and biosynthetic pathway of resveratrol in plants

### 2.1 Biosynthetic pathway of resveratrol

Resveratrol is a polyphenolic compound that was first isolated from the roots of *Polygonum cuspidatum* by Japanese scholar Takaoka in 1940 (Takaoka, 1940). Its chemical structure consists of two phenyl rings connected by an ethylene bridge, forming a 14-carbon skeleton. Naturally, it predominantly exists in the trans form, while the cis isomer is usually formed by the photoisomerization of trans-resveratrol under UV radiation or thermal stress (Berthet et al., 2019; Yoshinaga et al., 2025). Although cis-resveratrol is less abundant in nature, it also exhibits certain biological activities such as antioxidant and anti-inflammatory effects, and its content in some processed products (e.g., wine) may increase due to processing conditions (Zhang et al., 2013). The multiple phenolic hydroxyl groups confer strong antioxidant properties to resveratrol (Melchior and Kindl, 1991), enabling it to participate in the chemical defense of plants against ultraviolet radiation and pathogen stress (Pandith et al., 2019). Additionally, a study published in *Science* in 1997 demonstrated that resveratrol exerts anti-inflammatory and anticancer effects by inhibiting cyclooxygenase (COX) and peroxidase (POD), making it a promising candidate for cancer therapy (Jang et al., 1997).

**Figure 1:**
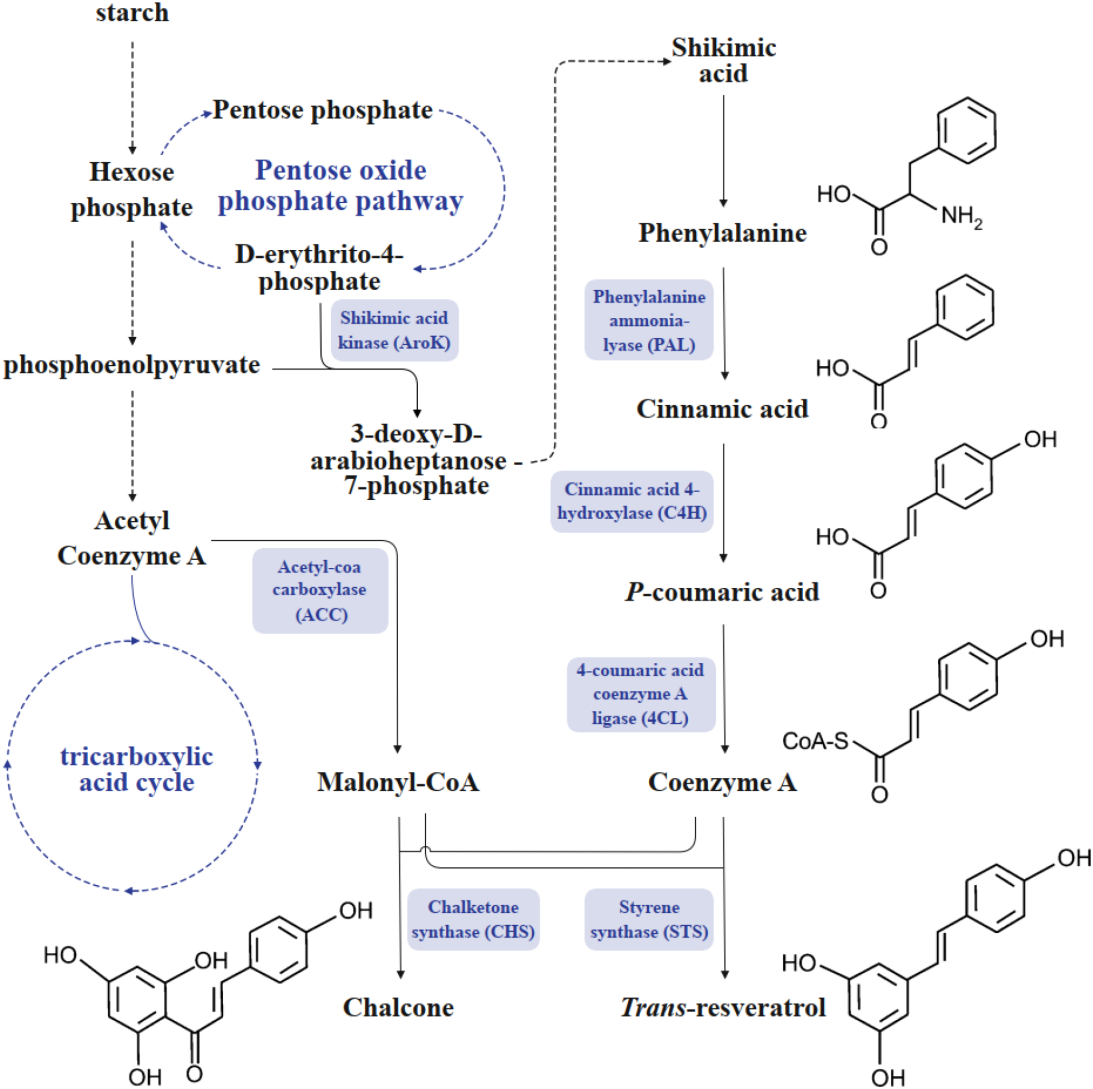
Biosynthetic pathway of trans-resveratrol.

The substrates for resveratrol biosynthesis originate from the shikimate pathway. The products of this pathway undergo a series of enzyme-catalyzed reactions to generate intermediates of the phenylpropanoid pathway, which serve as precursors for resveratrol synthesis as well as common substrates for other secondary metabolites such as flavonoids and tannins (Yu et al., 2005; Hammerbacher et al., 2011). The biosynthetic pathway of resveratrol is a branch of the phenylpropanoid metabolic pathway, specifically the stilbene biosynthetic pathway. In detail, the biosynthesis of resveratrol involves the following four key reactions: First, phenylalanine ammonia-lyase (PAL) catalyzes the deamination of L-phenylalanine to produce cinnamic acid. Subsequently, cinnamic acid is hydroxylated by cinnamate 4-hydroxylase (*C4H*) to form p-coumaric acid (Chen et al., 2006). Next, p-coumaric acid is activated by 4-coumarate: CoA ligase (*4CL*) to form the high-energy intermediate p-coumaroyl-CoA. Finally, stilbene synthase (*STS*) catalyzes the condensation of one molecule of p-coumaroyl-CoA with three molecules of malonyl-CoA, followed by decarboxylation to produce resveratrol (**Figure 1**) (Vannozzi, 2011).

It is noteworthy that the two substrates for resveratrol synthesis (p-coumaroyl-CoA and malonyl-CoA) can also be catalyzed by chalcone synthase (CHS) to generate chalcone through C6→C1 Claisen condensation (Schröder et al., 1988; Ferrer et al., 1999). This indicates a substrate competition between *STS* and CHS in plants. Both enzymes belong to type III polyketide synthases (PKS) (Jenke-Kodama et al., 2005). Structural analysis of proteins has shown that replacing key amino acids in the N-terminal active pocket of CHS (e.g., Thr132→Gly) can switch its function to *STS* activity (Austin et al., 2004). This suggests that *STS* and CHS may have originated from a common ancestor and divergently evolved to acquire distinct catalytic functions (product specificity).

### 2.2 Common transformations of resveratrol after biosynthesis

After biosynthesis in plants, resveratrol is commonly subjected to modifications and transformations by various enzymes, resulting in a diverse range of derivatives. These transformation processes not only increase the structural diversity of resveratrol but also significantly enhance its bioactivity and functional properties.

Firstly, under the catalysis of hydroxylase (such as hydroxylase), resveratrol can introduce an additional hydroxyl group (-OH) into the aromatic ring, forming resveratrol dihydroxy compounds (such as quercetin) (Murias et al., 2004). These hydroxylated products typically exhibit stronger antioxidant and antimicrobial properties.

Secondly, under the catalysis of glycosyltransferase, the hydroxyl group of resveratrol is glycosylated to form compounds such as resveratrol-3-O-β-glucoside (i.e., resveratrol glucoside) (Shimoda et al., 2015). Glycosylation increases the water solubility of resveratrol and enhances its stability in plant tissues, while also facilitating its storage in stress responses.

Additionally, resveratrol can be modified through methylation. Under the catalysis of S-adenosyl-L-methionine (SAM)-dependent methyltransferase (such as OMT), the hydroxyl group of resveratrol is converted to a methoxy group (-OCH_3_), forming methoxyresveratrol (such as pinosylvin) (Zhang and Go, 2007). Methylation enhances the lipophilicity and bioavailability of the molecule, significantly strengthening its antimicrobial and anti-inflammatory activities (Zhang and Go, 2007; Hongpin, 2011).

The hydroxyl group of resveratrol can also be prenylated by the catalysis of prenyltransferase (PT), resulting in prenylated products (Yang et al., 2016). This modification significantly increases its disease resistance and lipophilicity, enhancing its transport capacity in plants (Yang et al., 2018).

**Figure 2:**
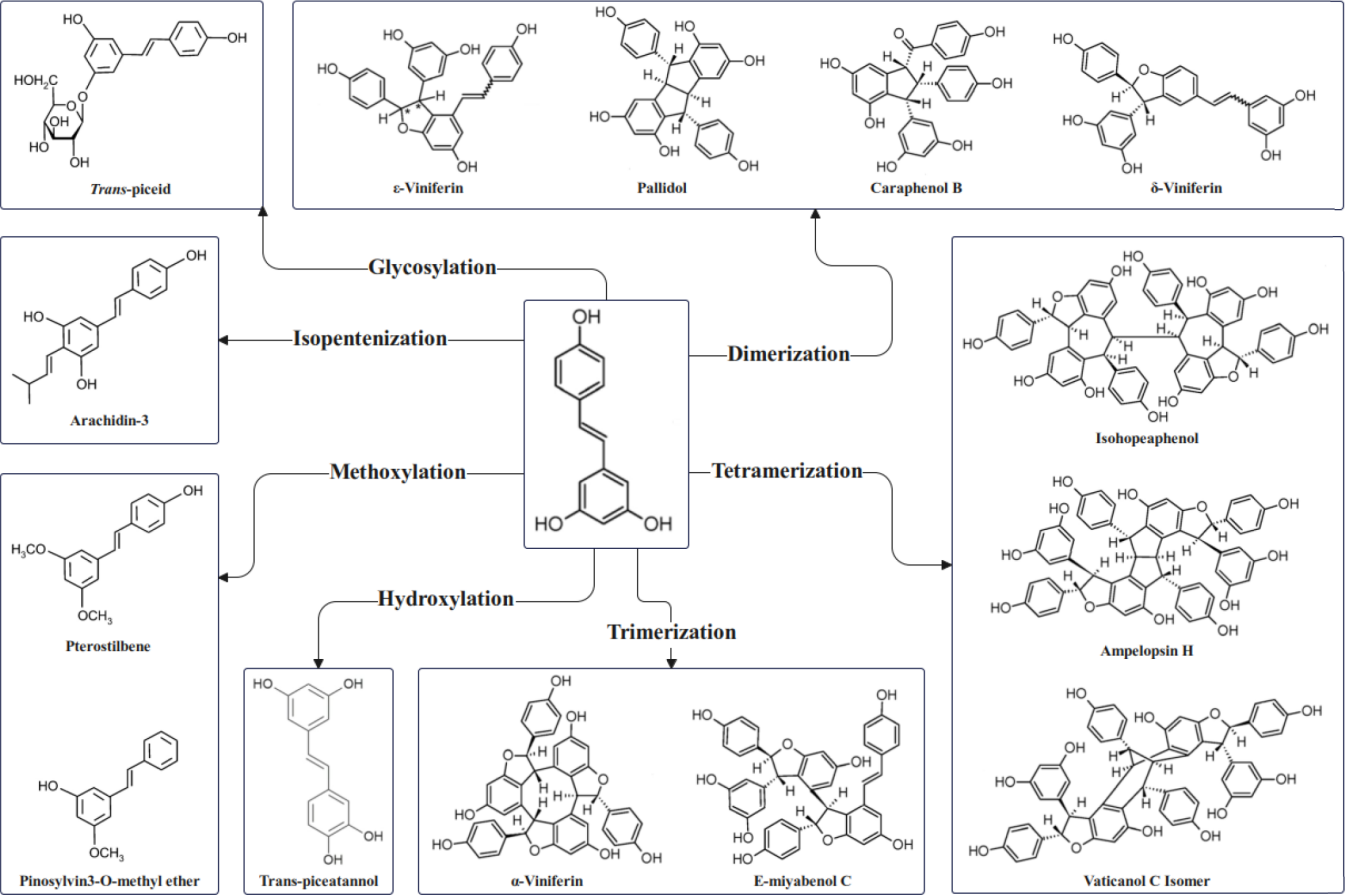
Common metabolic fates of resveratrol after biosynthesis.

Lastly, under the action of peroxidase (such as POX) or laccase, resveratrol undergoes oxidative coupling reactions to form dimers (such as *ϵ*-resveratrol dimer), tetramers, octamers, or higher oligomers (such as bis-resveratrol) (Yang et al., 2016). Oligomerized resveratrol derivatives usually exhibit stronger antioxidant properties (**Figure 2**) (Mikulski and Molski, 2010; Ismail et al., 2012).

## 3 Impact of elicitors on resveratrol biosynthesis

Under normal growth conditions, plants accumulate only trace amounts of resveratrol; however, its level rises sharply when the plant is exposed to biotic or abiotic stresses. These stress factors, collectively termed “elicitors,” are subdivided into biotic elicitors (e.g., pathogens) and abiotic elicitors (e.g., mechanical wounding, UV radiation, phytohormones, metal ions, and precursor molecules). Elicitors enhance resveratrol biosynthesis through multiple signaling cascades, and combinations of different elicitors frequently produce synergistic effects that further amplify the response. Here we focus on the regulatory roles of phytohormones and growth regulators, light quality, and metal ions in resveratrol biosynthesis (Versari et al., 2001; Billet et al., 2018).

### 3.1 Biotic elicitation of plant resveratrol biosynthesis

Pathogenic microorganisms (fungi and bacteria), endophytic fungi and bacteria, arbuscular mycorrhizal fungi (AMF), and plant-growth-promoting rhizobacteria (PGPR) all modulate the expression of key genes in the stilbene-biosynthetic pathway—most notably phenylalanine ammonia-lyase (PAL) and stilbene synthase (*STS*)—thereby inducing the accumulation of resveratrol and related stilbenes to varying extents.

Pathogenic fungi markedly stimulate the biosynthesis of resveratrol and its derivatives by activating the host defense network. *Plasmopara viticola* (downy mildew) and *Erysiphe necator* (powdery mildew) elicit pronounced accumulation of *trans-*resveratrol and its dimer *δ*-viniferin in grapevine leaves (Viret et al., 2018). After a 72-h challenge of lignified canes of the resistant cultivar *Solaris* with *P. viticola*, leaf *trans-*resveratrol content was ten-fold higher than in the susceptible cultivar *Chasselas*, while *δ*-viniferin reached 6.5-fold the level recorded in *Chasselas* (Pezet et al., 2015). Time-course transcriptional analyses further revealed a divergent expression pattern among stilbene-synthase (*STS*) family members in two-year-old rooted cuttings: 11 genes (including *VvSTS1* and *VvSTS2*) peaked at 24 h post-inoculation and subsequently declined, whereas *VvSTS14*, *VvSTS7* and *VvSTS8* displayed sustained up-regulation throughout the observation period.Interestingly, the moderately resistant cultivar *Canaiolo Nero* exhibited markedly higher *STS* transcriptional intensity than any other genotype tested (Ciaffi et al., 2019). Moreover, when grape cell-suspension cultures were challenged with *Botrytis cinerea*, *STS* transcript levels remained 2- to 3-fold above those of the control after 12 h, while intracellular resveratrol increased five-fold and the extracellular secretory pool surged 22.8-fold (Liswidowati et al., 1991). Infection by *Fusarium oxysporum* or *Trichoderma viride* likewise elicited a significant rise in foliar resveratrol biosynthesis in grapevine leaves (Sak et al., 2021).

Similarly, phytopathogenic bacteria can effectively elicit resveratrol biosynthesis in grapevine. Inoculation of *Vitis vinifera* cv. *Ugni Blanc* cuttings with *Pseudomonas syringae* pv. *pisi* raised leaf resveratrol to a maximum of 0.8 µg/g fresh weight (F.W.) at 48 h—an over five-fold increase relative to the initial 0.15 µg/g F.W.—followed by a decline to 0.44 µg/g F.W (Robert et al., 2001). Challenge with *Sphingomonas* sp. strain Hbc-6 likewise markedly enhanced the activities of several foliar antioxidant enzymes and significantly boosted resveratrol accumulation (Wang F. et al., 2022).Co-infection of 4-week-old *Vitis vinifera* cv. Chardonnay seedlings with *Agrobacterium tumefaciens* Gh1 (the grape crown-gall pathogen) and *Pantoea* sp. Sa14 for 10 d elevated stem resveratrol to 1.96 µg/g F.W.—seven-fold that of the non-inoculated control—whereas either bacterium applied individually yielded leaf resveratrol levels below those of the untreated plants (Asghari et al., 2020).

Endophytic fungi and bacteria markedly potentiate host secondary metabolism through metabolic crosstalk. Co-cultivation of *Rumex gmelini* Turcz with the endophytic *Aspergillus* sp. for 12 d elevated polydatin to 2.76 mg/g D.W.—23-fold that of the uninoculated control—while resveratrol itself increased 2.5-fold (Ding et al., 2018). Likewise, inoculation of *Polygonum cuspidatum* seedlings with the basidiomycete *Piriformospora indica* for 77 d raised leaf resveratrol and polydatin contents to 2.4 and 2.5 times the levels recorded in non-colonized plants, respectively, concomitant with a 2.5-fold up-regulation of *PcSTS* expression (Sun et al., 2022).Comparative evaluation of seven endophytic bacterial and six fungal isolates further identified *Biscogniauxia* sp. as the most potent elicitor: incubation of *Vitis amurensis* cell-suspension cultures with this fungus for 72 h raised the contents of all major stilbenes—including *trans-*resveratrol, polydatin, *δ*-viniferin and *ϵ*-viniferin—to 2–4-fold those of the non-inoculated control, outperforming every other microbial treatment. Concomitantly, transcript levels of phenylalanine ammonia-lyase (PAL) and stilbene synthase (*STS*) reached their highest values under this fungal challenge (Aleynova et al., 2021).

Endophytic fungi and arbuscular mycorrhizal fungi (AMF) markedly modulate the biosynthesis of resveratrol and its derivatives through root-mediated interactions. Inoculation of 30-day-old *Polygonum cuspidatum* Sieb. et Zucc. seedlings with *Funneliformis mosseae* for 77 days increased leaf resveratrol and polydatin contents to 3.2- and 1.54-fold those of the non-inoculated control, respectively, and up-regulated *PcSTS* expression five-fold (Sun et al., 2022).Similarly, 28-day-old *Polygonum cuspidatum* seedlings inoculated with *Funneliformis mosseae* for 84 days accumulated 0.51 mg/g polydatin in roots—3.4-fold that of non-mycorrhized plants—while *PcSTS* transcript abundance rose 3.2-fold (Wang et al., 2013). In another experiment, 21-day-old seedlings colonized by *Glomus mosseae* for 84 days exhibited doubled leaf resveratrol content without a statistically significant change in *PcSTS* expression (Deng et al., 2022). Likewise, root inoculation of 28-day-old *Vitis vinifera* plantlets with the plant-growth-promoting rhizobacterium *Paraburkholderia phytofirmans* PsJN maintained persistently elevated *PAL* and *STS* transcripts in roots across three sampling points, whereas in leaves a significant up-regulation was detected only at 7 days post-inoculation (Miotto-Vilanova et al., 2019).

### 3.2 Regulation of plant resveratrol biosynthesis by exogenous phytohormones and growth regulators

A substantial body of evidence demonstrates that exogenous phytohormones—including jasmonic acid and its derivatives (e.g., methyl jasmonate, MeJA), salicylic acid (SA), ethylene (ET), and melatonin (MT)—as well as growth-modulating agents such as methylated cyclodextrins, native cyclodextrins, and humic acids—act as potent elicitors capable of modulating the stilbene-biosynthetic pathway, thereby enhancing resveratrol accumulation in a wide range of plant species.

Methyl jasmonate (MeJA) is among the most powerful elicitors of resveratrol biosynthesis; it markedly up-regulates both phenylalanine ammonia-lyase (PAL) and stilbene synthase (*STS*) genes. In etiolated peanut seedlings and grape cell cultures, MeJA elevated transcript levels 3.2- and 5.4-fold, respectively (Pilaisangsuree et al., 2020; Yin et al., 2023). Co-treatments with cyclodextrin (CD) or Ca^2+^ produced clear synergies: combined MeJA and CD for 7 d increased *trans-*resveratrol content far beyond the levels achieved by either agent alone (Sabater-Jara et al., 2023; Yin et al., 2023), while a ternary combination of MeJA, UV-C and sucrose further amplified metabolite accumulation in transgenic grape cells (Wang et al., 2022b).

Salicylic acid (SA) applied alone similarly enhances resveratrol accumulation by up-regulating *PAL* and *STS* expression; 25–50 mg/L SA gives the maximal response in both grape cell cultures and peanut leaves (Xu et al., 2015a; Dai et al., 2016; Tyunin and Kiselev, 2017). When SA is combined with fungal-pathogen challenge, *VqSTS36* transcripts in grapevine are further elevated and disease resistance is strengthened (Yin et al., 2017), while SA plus Ca^2+^markedly increases resveratrol content in the roots of etiolated peanut seedlings (Wang et al., 2013).

Abscisic acid (ABA) stimulates the accumulation of both *cis-* and *trans-*polydatin in transgenic grape cells by up-regulating the ABA-responsive element binding factor gene *VvABF2* (Nicolas et al., 2014). Exposure of grape berries to ethylene elevates the concentrations of *trans-*resveratrol and its glucoside in the resulting wine (Becatti et al., 2014). Melatonin (MT) application significantly enhances resveratrol content in grape seeds, presumably by inducing phenylpropanoid-pathway genes and *STS* expression, thereby accelerating precursor supply for stilbene biosynthesis (Gao et al., 2024).

Biotic elicitors—including cyclodextrins (CDs), chitosan (CHT), sodium alginate, D-tagatose, glycyrrhizin, chito-oligosaccharides, and forchlorfenuron (CPPU)—also exert pronounced effects on resveratrol synthesis. CDs alone or combined with MeJA significantly elevate *trans-*resveratrol levels (Sabater-Jara et al., 2023). CHT activates the reactive oxygen species (ROS) signaling cascade; when applied together with MeJA or sodium alginate, it synergistically enhances both metabolite accumulation and the expression of defense-related genes (Xu et al., 2015b; Lucini et al., 2018; Chayjarung et al., 2022).Sodium alginate alone likewise up-regulates defense genes such as PAL and *STS*48 (Xu et al., 2015b). D-Tagatose increases phytoalexin content by enhancing PAL and *STS* transcript abundance (Mijailovic et al., 2022). Glycyrrhizin stimulates resveratrol-glycoside accumulation through modulation of ROS generation and cytoskeletal reorganization (Wang H. et al., 2021). Chito-oligosaccharides further amplify metabolite pools via induced expression of defense genes (Xu et al., 2015b). In contrast, forchlorfenuron (CPPU) negatively modulates the pathway: it represses *STS* and its upstream regulators *MYB*14/15, thereby suppressing stilbene biosynthesis (Tyagi et al., 2021).

### 3.3 Photoregulation of resveratrol biosynthesis by light quality

Light is a pivotal exogenous elicitor of resveratrol biosynthesis, with wavelength (light quality) exerting a decisive influence on the accumulation of resveratrol and other stilbenoids. Owing to its high energy, UV-C radiation is the most potent inducer. UV-C markedly enhances resveratrol content by up-regulating the expression of key phenylpropanoid-pathway genes (e.g., *STS* and PAL) (Paasela et al., 2023) and increasing the corresponding enzymatic activities (Pan et al., 2009).In developing grape berries, UV-C irradiation induces a stage-dependent accumulation of *STS* within secondary cell walls and chloroplasts (Pan et al., 2009). Independent work further shows that UV-C stimulates γ-aminobutyric acid (GABA) biosynthesis in grape, and GABA itself operates as a signalling metabolite that potentiates downstream stilbene production (Zhu et al., 2023). UV-B exposure likewise enhances grape resveratrol pools (Gai et al., 2022).

Combined treatments of ultraviolet radiation with other elicitors further amplify metabolite accumulation (Zhu et al., 2020). Co-application of UV-C and *p*-coumaric acid (CoA) elevated total stilbene content in spruce needles by 1.3-fold and significantly up-regulated transcription of *PjSTS1a* and *PjSTS1b* (Kiselev et al., 2019). Likewise, UV-C plus chitosan synergistically enhanced stilbene accumulation in grape cell-suspension cultures (Xu et al., 2016). A multifactorial treatment comprising UV-C, phenylalanine, salicylic acid and ultrasound increased resveratrol in germinated peanut to a maximum of 14.47 µg/g (Zhan et al., 2019).

Blue and red light also significantly promote stilbene accumulation in grape leaves by up-regulating the expression of key biosynthetic genes (*PAL, CHS*, *STS*) and concurrently suppress lesion development in detached-leaf assays (Ahn et al., 2015). In etiolated peanut sprouts, 48 h exposure to either blue or red light triples resveratrol content relative to dark controls (Li et al., 2021).

### 3.4 Regulation of resveratrol biosynthesis by metal ions

Metal ions are recognized as critical abiotic elicitors of resveratrol biosynthesis. FeCl_3_ foliar application significantly boosts resveratrol accumulation in grapevine, and this effect is positively correlated with the extent of leaf injury and reactive oxygen species (ROS) accumulation (Dong et al., 2017). Exogenous copper treatment not only enhances *trans-*resveratrol biosynthesis in peanut leaves (Wang, 2017)and seedling roots (Bao et al., 2021), but also stabilizes resveratrol-derived radicals through oxidative coupling, thereby promoting the formation of *δ*-viniferin, a resveratrol oligomer (Tamboli et al., 2012). In addition, aluminum treatment has been shown to induce the accumulation of *trans-*resveratrol and its glucoside in grape (Wang et al., 2022a). Other heavy metal ions, such as Co^2+^, Ag^+^, and Cd^2+^, significantly increase the accumulation of 3-O-glucosyl resveratrol (piceid) in grape cell suspension cultures (Cai et al., 2013).

### 3.5 Miscellaneous elicitors of plant resveratrol biosynthesis

In addition to the elicitor classes described above, exogenous supply of biosynthetic precursors and various environmental stresses have also been documented to enhance resveratrol production.

Phenylalanine spraying significantly elevates resveratrol content in etiolated peanut, although the increment remains lower than that achieved with copper treatment (Wang, 2017). Kiselev et al. demonstrated that p-coumaric acid not only improves the viability of grape cuttings under UV-C irradiation, but also amplifies the UV-C-triggered accumulation of resveratrol and other stilbenes; mechanistically, p-coumaric acid up-regulates a suite of stilbene-related genes, including those encoding resveratrol O-glucosyltransferase, polyphenol oxidase and cationic peroxidase (Kiselev et al., 2019).

Resveratrol-biosynthetic genes are also positively regulated by drought and waterlogging stresses (Baldi et al., 2024). Under prolonged drought, stilbene output—including resveratrol—rises markedly (Daldoul et al., 2024) via transcriptional activation of phenylalanine ammonia-lyase (*PAL*) and stilbene synthase (*STS*) (Sun et al., 2023). In the halophytic relative *Eutrema*, transcriptome profiling reveals significant enrichment of pathways committed to stilbenoids and diarylheptanoids (Ma et al., 2022).

Heat shock (40 °C) up-regulates *PAL*, *C4H* and *4CL* expression but fails to elevate resveratrol content significantly (Deng et al., 2017). Conversely, a combinatorial treatment of low temperature, ultrasound, UV, phenylalanine and salicylic acid on germinated peanut synergistically boosts resveratrol accumulation to 14.47 µg/g F.W.—substantially higher than any single-factor control (Zhan et al., 2019).

## 4 Transcriptional regulation of stilbene synthase (*STS*)-encoding genes

Most of our current knowledge on the transcriptional control of *STS* genes originates from Vitis spp. Investigations can be broadly grouped into two paradigms: first, direct activation or repression of *STS* promoters by individual transcription factors (TFs); second, combinatorial regulation arising from physical or functional interactions between TFs. To date, members of the *MYB*, *WRKY*, *bZIP*, Alfin-like, BSISTER, *ERF*, and *bHLH* families have been unequivocally implicated in modulating *STS* expression; the salient findings are summarized below.

*MYB* proteins were the first transcription factors (TFs) linked to stilbene-synthase (*STS*) regulation. Correlation analyses by *Holl* & *Vannozzi* revealed a robust positive relationship between the transcript levels of *VvMYB14/VvMYB15* and both *STS* expression and resveratrol accumulation; functional assays subsequently demonstrated that either *VvMYB14* or *VvMYB15* alone is sufficient to *trans-*activate the promoters of *VvSTS29* and *VvSTS41* (Höll et al., 2013). Importantly, *MYB*14 is itself post-transcriptionally controlled by microRNAs—*miR828* and *miR858* directly cleave *VvMYB14* mRNA, reducing its abundance and consequently dampening *STS* induction (Tirumalai et al., 2019). In a parallel layer of control, grape miR827a negatively regulates *VqMYB14*, thereby exerting an indirect repressive effect on *STS* transcription; the activity of the *MIR827a* promoter is, in turn, critically dependent on the ethylene-responsive factor *VqERF057* (Luo et al., 2024).

Further studies have demonstrated that additional *MYB*-type proteins—*VdMYB1* (Yu et al., 2019), *VqMYB35* (Wang and Wang, 2019), *VqMYB154* (Jiang et al., 2021) and *VqMYB15* (Luo et al., 2019; Wang et al., 2025)—act as positive regulators of distinct *STS* promoters, *trans-*activating *VdSTS2*, *VqSTS15*, *VqSTS28*, *VqSTS42*, *VqSTS46* and *VqSTS32*, respectively. In contrast, *VqMYB30* functions as a negative regulator that represses *VqSTS48* transcription and competes with activating *MYB* factors for occupancy of a common *cis-*element within the *VqSTS48* promoter (Mu et al., 2023).

**Figure 3:**
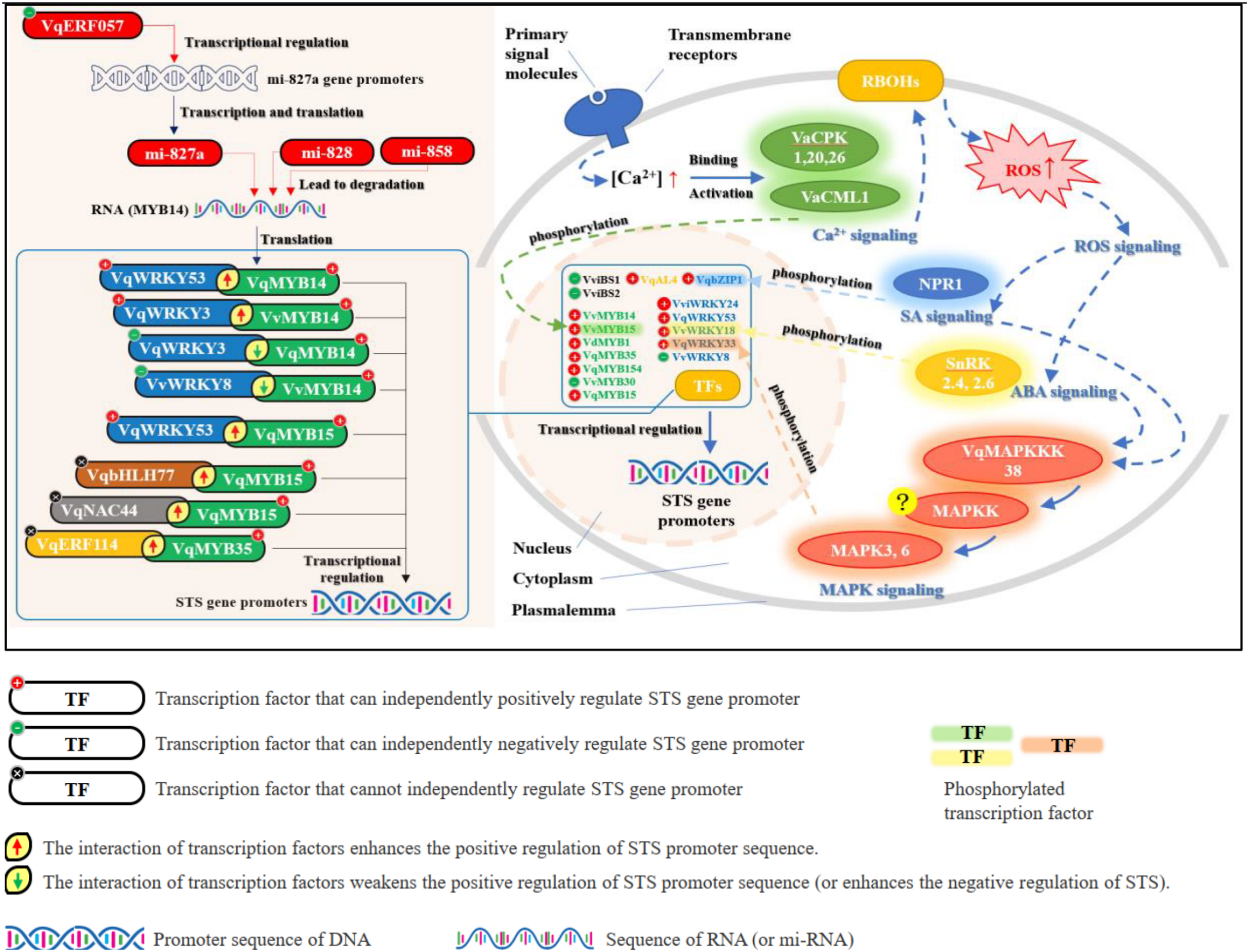
Proposed model of signalling transduction and transcriptional regulation governing resveratrol biosynthesis in plants. RBOHs, Respiratory Burst Oxidase homologs; ROS, Reactive Oxygen Species; CPK, Calcium-dependent Protein Kinases; CML, Calmodulin-like proteins; NPR, nonexpressor of pathogenesis-related genes; SnRK2, Sucrose non-fermenting 1-Related protein Kinase 2; MAPKKK, Mitogen-Activated Protein Kinase Kinase Kinase; MAPKK, Mitogen-Activated Protein Kinase Kinase; MAPK, Mitogen-Activated Protein Kinase; *STS*, Stilbene Synthase; *MYB*, *MYB* family transcription factor; *WRKY*, *WRKY* family transcription factor; *bHLH*, basic Helix-Loop-Helix family transcription factor; *NAC*, NAM, ATAF1/2, and CUC2 family transcription factor; *ERF*, Ethylene-Responsive Factor; *bZIP*, basic Leucine Zipper family transcription factor; AL, Alfin-like family transcription factor; *BSISTER*, Bsister family transcription factor; TF, transcription factor.

Beyond the *MYB* family, *WRKY* transcription factors (TFs) have emerged as key regulators of stilbene synthase (*STS*) gene expression. Several *WRKY* members function as positive regulators, including *VviWRKY24* (Vannozzi et al., 2018), *VqWRKY53* (Jiang et al., 2019), *VvWRKY18* (Wang K. et al., 2021), *VqWRKY31* (Yin et al., 2022), and *VqWRKY33* (Liu et al., 2023), all of which enhance *STS* transcription. Conversely, *VvWRKY8* acts as a negative regulator, suppressing *STS* expression (Jiang et al., 2019). Importantly, recent studies have revealed that *WRKY* and *MYB* proteins can physically interact to modulate STS transcription synergistically. For instance, *VviWRKY03* not only directly activates the promoter of *VviSTS29* on its own but also forms a protein complex with *VviMYB14*—a known positive regulator of *VviSTS29*. This interaction significantly enhances *VviMYB14*-mediated transcriptional activation of the *VviSTS29* promoter, providing a mechanistic basis for cooperative regulation of resveratrol biosynthesis by WRKY-*MYB* complexes (Vannozzi et al., 2018).*VqWRKY53* independently *trans-*activates *VqSTS32* and *VqSTS41*, and physically interacts with *VqMYB14* (or *VqMYB15*) to form a ternary complex that potentiates the activation of both *VqSTS32* and *VqSTS41* promoters beyond the level achieved by either *MYB* factor alone (Jiang et al., 2019). Conversely, the N-terminal domain of *VvWRKY8* associates with the N-terminal region of *VvMYB14*; this interaction sequesters *VvMYB14* from its target promoters, thereby abolishing its positive effect on *VvSTS15* and *VvSTS21* transcription (Jiang et al., 2019). In a parallel repressive module, *VvMYB30* not only directly suppresses *VvSTS15* and *VvSTS21* expression but also forms an N-terminal–mediated complex with *VvWRKY8*; this *MYB*–*WRKY* heterodimer reinforces the transcriptional repression of both *VvSTS15* and *VvSTS21* (Mu et al., 2023).Beyond the extensively characterized *MYB* and *WRKY* families, transcriptional control of *STS* genes has been extended to at least five additional TF families—*bZIP*, Alfin-like, BSISTER, *ERF* and *bHLH*. For example, *VqbZIP1* independently *trans-*activates the promoters of *VqSTS6*, *VqSTS16* and *VqSTS20* (Wang et al., 2019); the Alfin-like factor *VqAL4* enhances *VqNSTS4* transcription (Yan et al., 2023); and the BSISTER proteins *VviBS1* and *VviBS2* both activate *VviSTS48* expression on their own (Tang et al., 2025). Notably, two recent studies have uncovered an intriguing paradigm: certain TFs that are incapable of modulating *STS* promoters individually can still exert precise control over *STS* transcript levels via physical interaction with partner TFs, thereby forming heteromeric complexes that either potentiate or repress gene expression.For instance, *VqERF114* alone cannot modulate the transcription of *VqSTS15*, *VqSTS28*, *VqSTS42*, or *VqSTS46*; however, it physically interacts with *VqMYB35*—a positive regulator of these genes—to form a heteromeric complex that significantly potentiates *VqMYB35*-mediated *trans-*activation of their promoters (Wang and Wang, 2019). Similarly, *VqbHLH77* lacks intrinsic capacity to activate the *VqSTS48* promoter, yet its C-terminal domain forms a protein complex with *VqMYB15* via C–C interaction, thereby amplifying *VqMYB15*-dependent activation of *VqSTS48* transcription (Wang et al., 2025). Likewise, *VqNAC44* is incapable of independently regulating *STS* gene expression, but its C-terminal region interacts with the N-terminal domain of *VqMYB15*; this *VqNAC44*–*VqMYB15* complex enhances the *trans-*activation of *VqSTS9*, *VqSTS32*, and *VqSTS42*, ultimately accelerating resveratrol biosynthesis (**Figure 3**; **Tables 1**, **2**).

**Table 1:** Study on the direct regulation of *STS* promoters by transcription factor proteins.

Transcription factor family	Transcription factor name	Species name (cultivar name)	Gene to which the promoter sequence belongs	Regulation	Reference
MYB	*VvMYB14*	Vitis vinifera(Pinot Noir)	*VvSTS29, VvSTS41*	Positive regulation	(Höll et al., 2013; Fang et al., 2014)
	*VvMYB15*	Vitis vinifera(Shiraz)	*VvSTS29, VvSTS41*	Positive regulation	(Höll et al., 2013)
	*VdMYB1*	Vitis quinquangularis(Danfeng-2)	*VdSTS2*	Positive regulation	(Yu et al., 2019)
	*VqMYB35*	Vitis quinquangularis(Danfeng-2)	*VqSTS15, VqSTS28, VqSTS42, VqSTS46*	Positive regulation	(Wang and Wang, 2019)
	*VqMYB154*	Vitis quinquangularis(Danfeng-2)	*VqSTS9, VqSTS32, VqSTS42*	Positive regulation	(Jiang et al., 2021)
	*VvMYB30*	Vitis vinifera (Hongbaladuo)	*VvSTS15, VvSTS21*	Negative regulation	(Mu et al., 2023)
	*VqMYB15*	Vitis quinquangularis(Danfeng-2)	*VqSTS48*	Positive regulation	(Wang et al., 2025)
*WRKY*	*VviWRKY24*	The specific variety names are not indicated in the paper.	*VviSTS29*	Positive regulation	(Vannozzi et al., 2018)
	*VqWRKY53*	Vitis quinquangularis(Danfeng-2)	*VqSTS32, VqSTS41*	Positive regulation	(Wang et al., 2020)
	*VvWRKY18*	Vitis vinifera(Kyoho)	*VvSTS1, VvSTS2*	Positive regulation	(Wang K. et al., 2021)
	*VqWRKY33*	quinquangularis(Danfeng-2)	*VqNSTS3*	Positive regulation	(Liu et al., 2023)
	*VvWRKY8*	Vitis vinifera (Hongbaladuo)	*VvSTS15, VvSTS21*	Negative regulation	(Mu et al., 2023)
*bZIP*	*VqbZIP1*	Vitis quinquangularis(Danfeng-2)	*VqSTS6, VqSTS16, VqSTS20*	Positive regulation	(Wang et al., 2019)
Alfin-like	*VqAL4*	Vitis quinquangularis(Danfeng-2)	*VqNSTS4*	Positive regulation	(Yan et al., 2023)
BSISTER	*VviBS1, VviBS2*	Vitis vinifera(Thompson Seedless)	*VviSTS48*	Negative regulation	(Tang et al., 2025)
Alfin-like	At*MYB*44	Arabidopsis thaliana	At*STS*1	Positive regulation	(Wang et al., 2024)
*WRKY*	Pt*WRKY*22	Pinus tabuliformis	Pt*STS*3	Positive regulation	(Zhang et al., 2024)
*ERF*	*VqERF114*	Polygonum cuspidatum	*VqMYB35*	Positive regulation	(Wang and Wang, 2019)

**Table 2:** Direct regulation of *STS* promoters by protein-protein interactions.

Protein interaction category	Protein 1	Protein 2	Gene harboring the promoter sequence	Protein 1 alone regulates the target-gene promoter	Protein2 alone regulates the target-gene promoter	Protein–protein interaction and its resultant regulation of *STS* gene promoters	Experimental assays for protein–protein interaction	Reference
TF–TF interaction	*VviWRKY03*	*VviMYB14*	*VviSTS29*	Positive regulation	Positive regulation	*VviWRKY03* physically interacts with *VviMYB14* to form a heteromeric complex (the precise N- or C-terminal contact residues were not specified), thereby potentiating *VviMYB14*-mediated *trans-*activation of the *VviSTS29* promoter.	• Bimolecular fluorescence complementation assay	(Vannozzi et al., 2018)
TF–TF interaction	*VvWRKY8*	*VvMYB14*	*VvSTS15, VvSTS21*	No direct regulation	Positive regulation	The N-terminal domain of *VvWRKY8* directly interacts with the N-terminal region of *VvMYB14* to form a heteromeric complex that abolishes *VvMYB14*-mediated *trans-*activation of the *VvSTS15* and *VvSTS21* promoters.	• Yeast two-hybrid assay; • FRET-AB assays; • Bimolecular fluorescence complementation assay	(Jiang et al., 2019)
TF–TF interaction	*VqWRKY53*	*VqMYB14, VqMYB15*	*VqSTS32, VqSTS41*	Positive regulation	Positive regulation	*VqWRKY53* physically interacts with *VqMYB14* (or *VqMYB15*) to form a heteromeric complex (the precise N- or C-terminal contact residues were not specified), thereby enhancing *VqMYB14*- (or *VqMYB15*-) mediated *trans-*activation of the *VqSTS32* and *VqSTS41* promoters.	• Yeast two-hybrid assay; • Bimolecular fluorescence complementation assay	(Jiang et al., 2019)
TF–TF interaction	*VvMYB30*	*VvWRKY8*	*VvSTS15, VvSTS21*	Negative regulation	Negative regulation	*VvMYB30* alone acts as a repressor of *VvSTS15* and *VvSTS21* transcription. Its N-terminal domain directly interacts with the N-terminal region of *VvWRKY8* to form a heteromeric complex that reinforces *VvWRKY8*-mediated negative regulation of the *VvSTS15* and *VvSTS21* promoters. Moreover, this *VvMYB30*–*VvWRKY8* interaction, together with their concurrent binding to the *STS15/21* promoter sequences, competitively excludes *VvMYB14*, thereby blocking the positive activation normally exerted by *VvMYB14* on the *VvSTS15* and *VvSTS21* promoters.	• Yeast two-hybrid assay; • Bimolecular fluorescence complementation assay; • GST pull-down assay	(Mu et al., 2023)
TF–TF interaction	*VqERF114*	*VqMYB35*	*VqSTS15,* *VqSTS28,* *VqSTS42,* *VqSTS46*	No direct regulation	Positive regulation	*VqERF114* physically interacts with *VqMYB35* to form a heteromeric complex (the precise N- or C-terminal contact residues were not specified), thereby enhancing *VqMYB35*-mediated *trans-*activation of the *VqSTS15*, *VqSTS28*, *VqSTS42* and *VqSTS46* promoters.	• Yeast two-hybrid assay; • Bimolecular fluorescence complementation assay	(Wang and Wang, 2019)
TF–TF interaction	*VqbHLH77*	*VqMYB15*	*VqSTS48*	No direct regulation	Positive regulation	The C-terminal domain of *VqbHLH77* directly interacts with the C-terminal region of *VqMYB15* to form a heteromeric complex that potentiates *VqMYB15*-mediated *trans-*activation of the *VqSTS48* promoter.	• Yeast two-hybrid assay; • Bimolecular fluorescence complementation assay; • GST pull-down assay	(Wang et al., 2025)
TF–TF interaction	*VqNAC44*	*VqMYB15*	*VqSTS9,* *VqSTS32,* *VqSTS42*	No direct regulation	Positive regulation	*VqNAC44* alone is unable to modulate STS transcription; however, its C-terminal domain interacts with the N-terminal region of *VqMYB15* to form a heteromeric complex that enhances *VqMYB15*-mediated positive regulation of *VqSTS9*, *VqSTS32*, and *VqSTS42* transcription.	• Yeast two-hybrid assay; • Bimolecular fluorescence complementation assay	(Wang et al., 2024)
Signaling pathway Kinase phosphorylation of TFs	*VqbZIP1*	*VqSnRK2.4,* *VqSnRK2.6*	*VqSTS6,* *VqSTS16,* *VqSTS20*	Positive regulation	Not specified in the original paper	The kinases *VqSnRK2.4* and *VqSnRK2.6* directly phosphorylate *VqbZIP1*, thereby enhancing *VqbZIP1*-mediated *trans-*activation of the *VqSTS6*, *VqSTS16*, and *VqSTS20* promoters.	• Yeast two-hybrid assay; • Bimolecular fluorescence complementation assay	(Wang et al., 2019)
Signaling pathway Kinase phosphorylation of TFs	*VvWRKY18*	*VvNPR1*	*VvSTS1,* *VvSTS2*	Positive regulation	Not specified in the original paper	Although *VvNPR1* lacks intrinsic DNA-binding activity, it physically interacts with the STS-positive transcription factor *VvWRKY18* to form a heteromeric complex (the precise N- or C-terminal contact residues were not specified). This association potentiates *VvWRKY18*-mediated *trans-*activation of the *VvSTS1* and *VvSTS2* promoters.	• Yeast two-hybrid assay; • GST pull-down assay • Co-immunoprecipitation assay	(Wang K. et al., 2021)
Signaling pathway Kinase phosphorylation of TFs	*VqWRKY33*	*VqMAPK3, VqMAPK6*	*VqNSTS53*	Positive regulation	Not specified in the original paper	The kinase *VqMAPK3* (or *VqMAPK6*) directly phosphorylates Ser residues within the SP-cluster motif of *VqWRKY33*, thereby enhancing *VqWRKY33*-mediated *trans-*activation of the *VqNSTS53* promoter.	• Yeast two-hybrid assay; • GST pull-down assay; • Co-immunoprecipitation assay	(Liu et al., 2023)

## 5 Signal-transduction cascades underlying resveratrol biosynthesis

Upon perception of abiotic or biotic elicitors (UV-C, pathogens, wounding, heavy metals, etc.), plant cells convert the primary physical signal into rapid electrical and/or ion-flux events at the plasma membrane (Garcia-Brugger et al., 2006). The resultant transient elevations of cytosolic Ca^2+^ ([Ca^2+^]cyt) and apoplastic/cytosolic reactive oxygen species (ROS) operate as the two archetypal second messengers that initiate downstream signalling (Ma et al., 2013). Acting as central hubs, Ca^2+^and ROS activate calmodulin-like proteins (CMLs) and calcium-dependent protein kinases (CDPKs), modulate hormone-dependent branches, and prime mitogen-activated protein kinase (MAPK) cascades, ultimately channelling the information flow toward the stilbene-biosynthetic machinery.

Functional characterization of calcium sensors has largely relied on transgenic over-expression in grape suspension cells. Among CDPKs, *VaCPK1*—rapidly induced by drought, cold and heat (Dubrovina et al., 2013)—boosts *VaSTS1*, *VaSTS2* and *VaSTS4* transcripts 35-, 15- and 12-fold, respectively, and increases resveratrol 1.7–4.6-fold under non-stress conditions (Aleynova et al., 2017). Analogously, *VaCPK26* over-expression elevates *VaSTS1*, *VaSTS2* and *VaSTS4* mRNA 60-, 45- and 20-fold, leading to a 2.5–6.2-fold rise in resveratrol (Aleynova et al., 2017), whereas *VaCPK20* specifically up-regulates *VaSTS7* in *V. amurensis* cells (Dubrovina et al., 2015). By contrast, *VaCPK9* over-expression suppresses stilbene biosynthesis during drought (Dubrovina et al., 2016), and although *VaCPK3a* is highly expressed in young leaves, its transcript level correlates negatively with basal resveratol content (Kiselev et al., 2013; Dubrovina et al., 2016); ectopic over-expression of *VaCPK3a* fails to alter stilbene titres (Dubrovina et al., 2016). Independently, over-expression of the calmodulin-like gene *VaCML1* enhances PAL and *STS* expression and enzyme activity, resulting in elevated resveratrol even in the absence of external stimuli (Aleynova et al., 2022).

Despite the extensive documentation of exogenous phytohormones and biotic elicitors on resveratrol induction, the molecular circuitry through which endogenous ABA, JA, SA or ET signalling is transduced into *STS* activation remains largely uncharted. Over-expression of canonical hormone-pathway genes and their consequent effects on *PAL*/*STS* expression or metabolite titres have rarely been interrogated. Nevertheless, several recent breakthrough studies have begun to establish protein–protein intersections that physically link hormone signalling cascades to *STS* transcriptional hubs, and to dissect the signal cross-talk between hormonal and MAPK modules during resveratrol biosynthesis.

In *Vitis vinifera*, the salicylic-acid (SA) hub protein *VvNPR1* physically interacts with and activates the *WRKY* transcription factor *VvWRKY18*; the resulting heterocomplex specifically binds to W-box *cis-*elements in the *VvSTS1/2* promoters. Following SA treatment, the promoter-binding efficiency of the complex increases 2.3-fold, elevating *STS* transcript levels by 3.5-fold and boosting resveratrol accumulation (Wang K. et al., 2021).

In the Chinese wild grape *V. quinquangularis*, the stress-activated kinases *VqSnRK2.4* and *VqSnRK2.6* phosphorylate the bZIP transcription factor *VqbZIP1* at Ser-217. This post-translational modification enhances *VqbZIP1* binding to ABRE motifs in the promoters of *VqSTS6*, *VqSTS16*, and *VqSTS20*. Over-expression of either SnRK2 gene increases *STS* transcription 3.2-fold and raises resveratrol content to 1.8-fold that of wild-type plants (Wang et al., 2019).

MAPK cascades provide an additional layer of integration. *VqMAPKKK38*-over-expressing grape calli treated with SA, JA, or ABA exhibit a 3.8-fold increase in *STS* expression and a 2.1-fold rise in resveratrol content relative to controls; RNA-interference-mediated silencing of *VqMAPKKK38* reduces resveratrol levels to 32% of the control value (Jiao et al., 2017).

Very recently, VqMAPK3 and VqMAPK6 have been shown to phosphorylate Vq*WRKY*33 at the Thr-152 site. This phosphorylation modification enhances the binding affinity of Vq*WRKY*33 to the promoter region of VqN*STS*53, thereby markedly strengthening its trans-activation activity and further promoting stilbene biosynthesis (Liu et al., 2023). A latest study by Liu et al. (2023) further confirmed that this regulatory module is conserved in other plant species and participates in the response to multiple abiotic stresses (Liu et al., 2023).

## 6 Conclusions and perspectives

The stilbenoid pathway leading to resveratrol is now largely resolved at the biochemical level. UV radiation, pathogenic microbes, exogenous phytohormones (e.g., MeJA, SA), abiotic stresses (drought, salinity) and heavy-metal exposure consistently enhance resveratrol accumulation across plant species. Stilbene synthase (*STS*), the rate-limiting step, is transcriptionally gated; its expression is directly proportional to resveratrol output. *MYB* TFs are the master switches, but their activity is fine-tuned by physical interactions with *WRKY*, *ERF, NAC* and other TF families. Calcium relays (*CMLs, CDPKs*) and hormone-activated kinases (*SnRK2, NPR1, MAPKs*) further modulate these complexes via phosphorylation, thereby amplifying or attenuating *STS* transcription. Despite these advances, two key knowledge gaps remain to be addressed.

First, whether members of TF families outside the *MYB*/*WRKY* clade can directly engage *STS* promoters through protein–protein interactions is still unexplored, and future studies should focus on identifying novel TF families involved in *STS* regulation to expand the current regulatory network.

Second, in JA and ET signalling cascades, the identity of kinases that might phosphorylate *STS*-associated TFs and the consequent impact on resveratrol output await systematic investigation, which will help clarify the cross-talk between hormone signalling and *STS* transcription. Looking forward, with the development of single-cell transcriptomics, spatial metabolomics and gene editing technologies, future research can further refine the regulatory mechanisms of resveratrol biosynthesis at the single-cell level, and realize the precise improvement of resveratrol content in different plant species through synthetic biology strategies.

In addition, exploring the regulatory patterns of resveratrol biosynthesis in non-model plants will provide new insights for the development of novel plant-derived medicinal resources, promoting the application of resveratrol in the fields of medicine, health care and agriculture.
